# Recent Progress of Nanodiamond Film in Controllable Fabrication and Field Emission Properties

**DOI:** 10.3390/nano13030577

**Published:** 2023-01-31

**Authors:** Xin Guo, Yajun You, Aida Bao, Pinggang Jia, Jijun Xiong, Junshuai Li

**Affiliations:** 1 National Key Laboratory for Electronic Measurement Technology, North University of China, 3 Xueyuan Road, Taiyuan 030051, China; 2 Key Laboratory of Special Function Materials and Structure Design of the Ministry of Education, Lanzhou University, 222 South Tianshui Road, Lanzhou 730000, China

**Keywords:** nanodiamond film, field emission properties, fabrication methods, grain size/phase controlling, doping engineering

## Abstract

The interest in the field electron emission cathode nanomaterials is on the rise due to the wide applications, such as electron sources, miniature X-ray devices, display materials, etc. In particular, nanodiamond (ND) film is regarded as an ideal next-generation cathode emitter in the field emission devices, due to the low or negative electron affinity, small grain size, high mechanical hardness, low work function, and high reliability. Increasing efforts are conducted on the investigation of the emission structures, manufacturing cost, and field emission properties improvement of the ND films. This review aims to summarize the recent research, highlight the new findings, and provide a roadmap for future developments in the area of ND film electron field emitter. Specially, the optimizing methods of large-scale, high-quality, and cost-effective synthesis of ND films are discussed to achieve more stable surface structure and optimal physical properties. Additionally, the mainstream strategies applied to produce high field emission performance of ND films are analyzed in detail, including regulating the grain size/boundary, hybrid phase carbon content, and doping element/type of ND films; meanwhile, the problems existing in the related research and the outlook in this area are also discussed.

## 1. Introduction

Nanodiamond (ND) films are normally composed of a mixture of sp^3^ and sp^2^ hybridized carbon, remains most of the excellent properties of the bulk diamond, especially including the high mechanical hardness [1], increased wear resistance [2], chemical stability [3], low coefficient of thermal expansion [4], wide optical transparency [5], and good biocompatibility [6]. Furthermore, the ND films possess unique physical and chemical properties, especially the higher volume density of grain boundaries, the low surface roughness, the more sp^2^ hybridized carbon concentration, and the low work function value [7,8,9]. These properties make them possible as an electron emission cathode material for the vacuum microelectronic devices and field emission devices [10,11,12]. However, the wide band gap energy (∼5.5 eV), and the compact crystal structure of the bulk diamond affect the complement transfer of electrons, and the formation of the depletion region in diamond, thus hindering the industrial application in electronic and optoelectronic devices [13,14].

During the last decades, many efforts, such as the N/B element doping [15,16,17], the surface morphology [18,19], the micropatterned structures [20,21], and the introduction of the high content sp^2^ hybridized carbon [22,23], were conducted to obtain a low surface potential barrier and a low work function of the ND films, then substantially improves the field emission properties. For example, Hao T. et al. reported that the ND cones synthesized by the gray-scale patterns with a focused-ion-beam (FIB) system could obtain a high emission current up to 54 μA at an applied voltage of 10 V [21]. Guo X. et al. proposed a N-doped ND film by heating the solid thin layer of urea in a resistance-heating furnace, exhibiting a low turn-on electric field strength of 3.6 V µm^−1^ defined at the current density of 0.01 mA cm^−2^ [15]. Moreover, the field emission properties are largely determined by the quality and microstructure of synthesized ND films. Many production methods are proposed, such as chemical vapor deposition with various source, laser irradiation, and assembling detonated ND particles with various solutions [24,25]. Recently, Hong S.P. reported a unit combination of three plasma sources to synthesize even distributed diamond grains with a diameter of 0.1–1 μm, which was benefit for achieving a large-scaled diamond film with high crystallinity [26]. However, there still exists many discordant sounds that it is difficult to simultaneously obtain large-scaled, high quality, smooth ND films using current production methods. More advanced and cost-effective method of the ND films needs to be explored.

**Figure 1:**
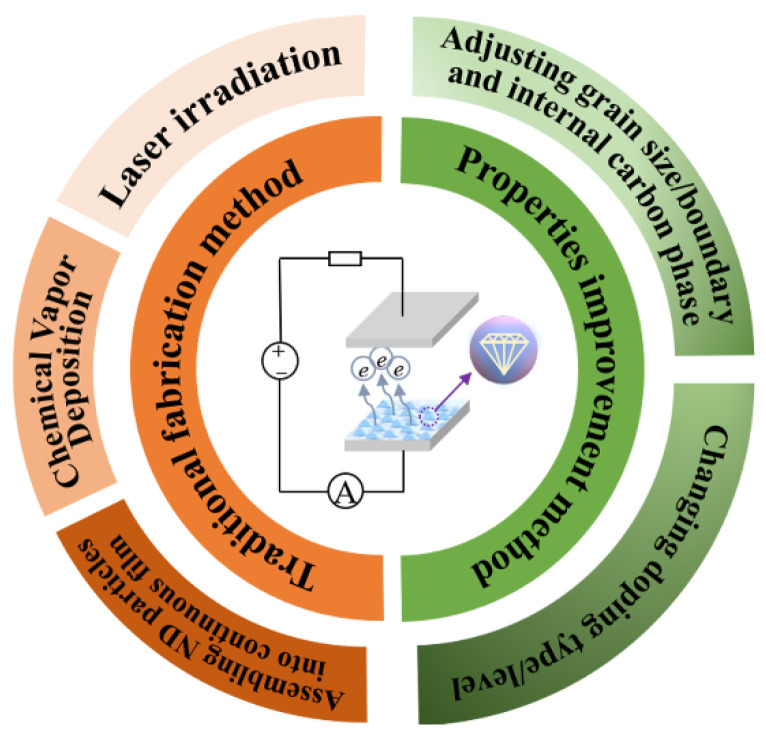
Fabrication and field emission properties improvement methods of the nanodiamond (ND) films.

In this paper, recent progresses of the ND films (excluding ultra-ND films) in the controllable fabrications and the field emission properties are reviewed, as shown in Figure 1. The preparation methods of ND film are summarized including the chemical vapor deposition, assembling ND particles into continuous film, and laser irradiation, and the growth factors and nucleation process of various methods ND films are analyzed. In addition, the improvement methods of field emission property, such as adjusting the grain size/boundary, the concentration of sp^2^ phase, and doping type are especially introduced. 

## 2. Fabrication Method and Nucleation Process of Nanodiamond Film

These ND films are commonly synthesized via chemical vapor deposition (CVD) and diamond seeding process, assembling NDs into continuous films, laser irradiation, and other methods. As the most important method, CVD and diamond seeding process has unparalleled advantage in controlling the grain sizes, defect structures and concentrations, and mechanical strength, and electrical conductivity properties of ND film. However, due to the high growth temperature (~700 °C), and the high requirement of vacuum environment, the growth substrates and the equipment of CVD method are severely restricted the large-scale production. Assembling ND particles into continuous film is another valuable preparation method, in which the size and purity of ND particles in film can be well controlled. Other methods include confined laser irradiation [27,28], bias-enhanced growth [29], and electrophoretic deposition (EPD) combined with annealing process [30], etc.

### 2.1. Chemical Composition and Structure of Nanodiamonds

ND films consist of ND grains of the order of 10–100 nm in size, surrounded by a carbon film with complex composition. The film composition and the linked bonding are dependent on the fabrication methods. Additionally, the chemical and physical properties of ND film is mainly determined by the chemical composition and inherent structure of NDs, including the average grain size and distribution of NDs, the sp^2^/sp^3^ hybridized carbon ratio, etc. [31]. The shape of NDs is normally regarded as spherical. However, more experimental high-resolution transmission electron microscopy images revealed that the NDs is randomly different shape clusters with different sizes [32]. Recent nuclear magnetic resonance reports suggested that each ND particle was consisted of a sp^3^ bonded carbon diamond core with a diameter of 2–3 nm, an ultra-thinly nonhomogeneous translational intermediate fullerene-like sp^2^ carbon shell, and a surface graphitic carbon layer [33,34,35,36]. The diamond core is the primary structural feature of NDs that distinguishes NDs from other carbonaceous materials, which is responsible for the high refractive index of NDs (~2.4) and strong light scattering. More specially, the properties of NDs can be adjusted further by introducing particular defects (i.e., dopants/impurities) into diamond lattice, and changing the host nanocrystal size [37,38,39]. The surface graphitic carbon layer is always bounded with hydrogen, oxygen, or functional groups for stabilization of the structure. Particularly, the σ dangling bonds on the surface carbon layer of NDs are unstable, strongly influences the electronic structure due to the dominant role of surface on nanoscale. Thus, the surface of NDs is easily modified or reconstructed, then leading to an extreme diversity of electronic properties. For example, after hydrogenation modification of the detonation NDs, the presence of surface states (σ_s_^1^σ_p_^2^π^1^) without overlapping of π levels were conducted by Belobrov P.I. et al. [40]. Kaciulis S. et al. also verified that the valence band of diamond is significantly modified by the hydrogenation modification [41].

### 2.2. Chemical Vapor Deposition (CVD)

#### 2.2.1. Diamond Seeding Process

**Figure 2:**
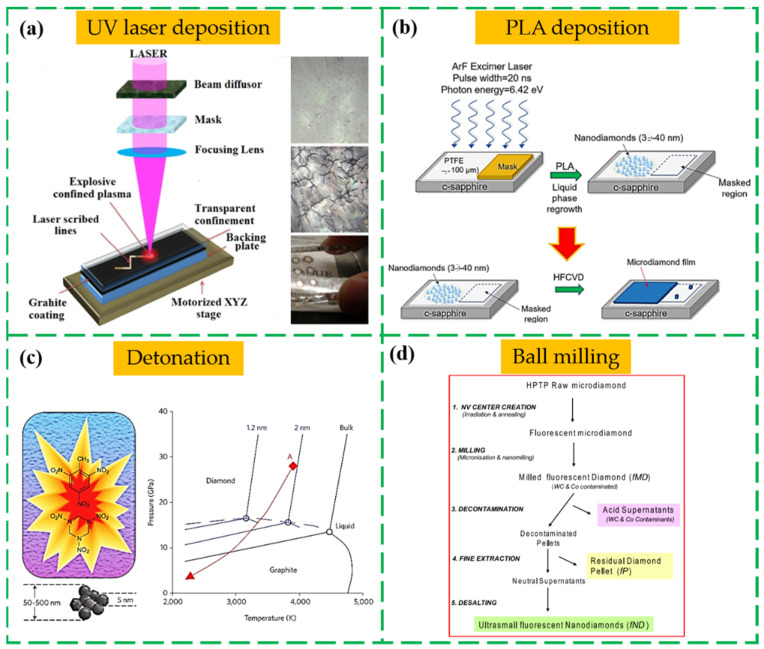
The commercially methods of the ND particles used in seeding process. The experimental schematic process of (**a**) the UV laser deposition [27], (**b**) PLA deposition [50], (**c**) detonation [51], and (**d**) ball milling technique [53].

Before synthesizing the ND films by CVD, a diamond seeding process is a critical step to essentially enhance the nucleation quality and smoothness of ND film in non-carbide forming substrates [42,43,44]. Many seeding procedures are employed for the ND film growth, including mechanical abrasion method with a diamond grit, spin-off coating method on the substrate with a diamond-containing particles, printing method of seeds by a stamp brought in contact with the substrate, ultrasonic treatment method in a suspension of diamond powder, and bias-enhanced nucleation (BEN) deposition of diamond on a substrate [45,46,47,48,49,50,51,52]. The quality of ND film is directly dependent on the seeding parameter, such as the density and size distribution of ND particles. Normally, the ND particles as a seeding layer are availably synthesized by intense ultraviolet (UV) laser irradiation [27], pulsed laser annealing (PLA) [50], detonation techniques [51], ball milling process of high pressure and high temperature (HPHT) diamond microcrystals [53], and CVD with hot filament, microwave, and plasma-assisted energy sources [54,55,56]. The majority of used commercially approaches rely on laser deposition, detonation, and ball milling technique, as shown in Figure 2a–d.

#### 2.2.2. CVD with Different Energy Sources

The ND film was first synthesized by CVD technology and was named as ‘nanocrystalline diamond’ at the Workshop on the Science and Technology of Diamond Thin Films in 1990 [57]. It is reported that the CVD ND films normally consist of ND grains with columnar internal structure [24]. Specifically, the CVD growth process of ND films begins with high-density nucleation to forms nanodiamond domains, and then grows in a columnar manner. The film morphology and internal structure are usually determined by the growth conditions and the CVD energy sources. There are many CVD methods with different energy sources used for facilitating the synthesis of ND films, such as pulsed microwave plasma [57,58], direct current (DC) glow discharge plasma [59,60], inductively coupled radio frequency (RF) plasma [61,62], and hot filament [63,64]. Among them, the plasma enhanced CVD methods using various plasma energy sources to generate plasma are effective for reducing the growth temperature and expanding the selection range of substrates. 

**Figure 3:**
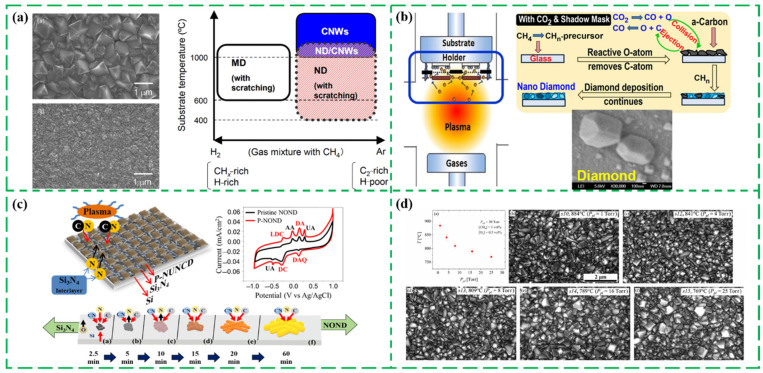
The synthesized ND films via microwave plasma CVD. (**a**) The SEM images of microdiamond film and ND films, and the corresponding variation of the samples as functions of substrate temperature and gas composition [69]. (**b**) Schematic diagram of the active plasma zone and the growth process of the low-temperature nano-/microcrystalline diamond growth [70]. (**c**) The formation evolution process and the CV scans of the ND film [71]. (**d**) The relationship curves between the average substrate temperature and the plenum pressure *P*_pl_, and the SEM images of the corresponding samples [72].

Moreover, the microwave plasma (MW) CVD is widely used to synthesize ND films on various chemically dissimilar surface substrates owing to the relatively low growth temperature, which utilizes the microwave energy to heat and decompose the gas molecules in the cavity into reactive groups, then finally obtain a high-quality ND film [65,66,67,68,69,70]. For example, Cheng C.Y. et al. prepared the ND films and microdiamond films via a MW plasmas method in Figure 3a. Additionally, it was suggested that the growth quality of ND film was restricted by the substrate surface pretreatment condition (scratching and seeding) rather than gas-phase condition [69]. Das D. et al. successfully synthesized the ND films and even microdiamond films on glass substrates at the temperature of ∼300 °C using CO_2_/CH_4_/H_2_ and provided a specific shadow-mask assembly to promote the nucleation of the diamond species and the diffusion growth of the nano-/micro diamond network on the untreated glass substrates, as shown in Figure 3b [70]. The CO_2_ was introduced as the supplementary gas to eliminate the amorphous carbon component in the synthesized nano-microdiamond. A three-dimensional patterned nitrogen-incorporated ovoid-shaped nanodiamond (NOND) was manufactured by Chang C. et al. to further improve the sensing properties. Additionally, the growth process of NOND film was investigated, including the initial stages of the diffusion of nitrogen atoms into the Si3N4/plasma interface, and the subsequent deposition of the NOND film, as shown in Figure 3c [71]. Giussani A. et al. synthesized the ND films using the diamond seeding process and MWCVD technology in Figure 3d, and the chamber pressure and the substrate temperature were critical to the induction time and the growth rate of ND films [72]. 

**Figure 4:**
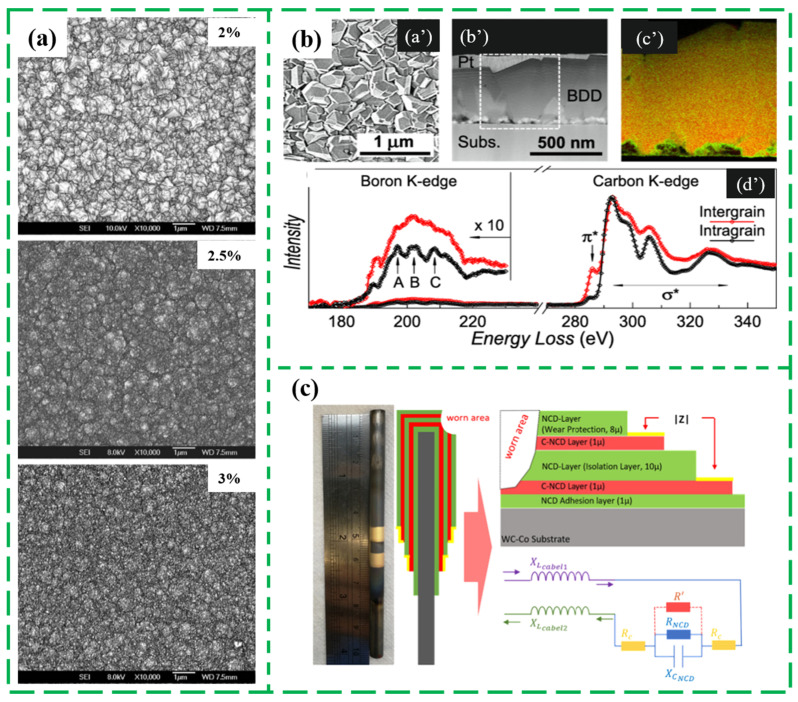
The synthesized ND film via hot filament CVD. (**a**) The SEM images of the synthesized ND films under different carbon concentration ratio [63], (**b**) the SEM image (**a’**), cross section STEM image (**b’**), STEM-EELS image of boron (in green) and carbon (in red) distributions in the white dashed window of STEM image (**c’**), and the characteristic EELS spectra (**d’**) recorded from the intragrain and intergrain regions of the heavily boron-doped nanodiamond films [74], (**c**) a nanocrystalline diamond multilayer system including two conductive nanocrystal-line diamond layers and one non-conductive nanocrystalline diamond [75].

In the contrast, the hot-filament (HF) CVD method is considered as the most convenient and simple method to synthesize nanodiamond films under relatively high-temperature, which uses the high temperature produced by the hot filament to pyrolyze the carbon-containing gas into active groups [73]. For example, Su Q. et al. synthesized the ND films via HFCVD and analyzed the effects of carbon concentration on the ND structure film [63]. As the carbon concentration ratio in the total gas increased, the ND grain size, the film roughness and the inter sp^3^ carbon phase concentration decreased while the sp^2^ carbon phase increased in nano-diamond films in Figure 4a. Additionally, a heavily boron (B)-doped ND thin films was also fabricated by the HFCVD, which can be converted from a superconductor to an insulation by the pressure driven as shown in the SEM/cross section STEM image and the characteristic EELS spectra recorded from the intragrain and intergrain regions of the heavily boron-doped nanodiamond films (Figure 4b), which was attributed to the suppression of the Josephson intergrain coupling between the superconducting nanodiamond grains [74]. So far, the HFCVD technology is still a popular approach to fabricate the ND films due to the relatively low equipment cost and simple process. In 2022, a nanocrystalline diamond multilayer system including two conductive nanocrystalline diamond layers and one non-conductive nanocrystalline diamond produced via HFCVD coating process with CH_4_/H_2_ mixture gas was applied in a wear sensor prototype as exhibited in Figure 4c [75].

The growth parameters using CVD with different energy source are summarized in Table 1. It can be concluded that the obtained ND films from various CVD method are normally grown in a H_2_-rich, carbon-containing gas-lean mixture atmosphere under growth substrate temperature from 250 °C to 1200 °C [59,62,64,69,70,71,72,73,74,75]. Presumably the higher ratio of CH_4_ in H_2_, the more non-diamond carbon incorporation quantity, and the smaller grain size of ND particle in ND film.

#### 2.2.3. Assembling ND Particles into Continuous Films

**Figure 5:**
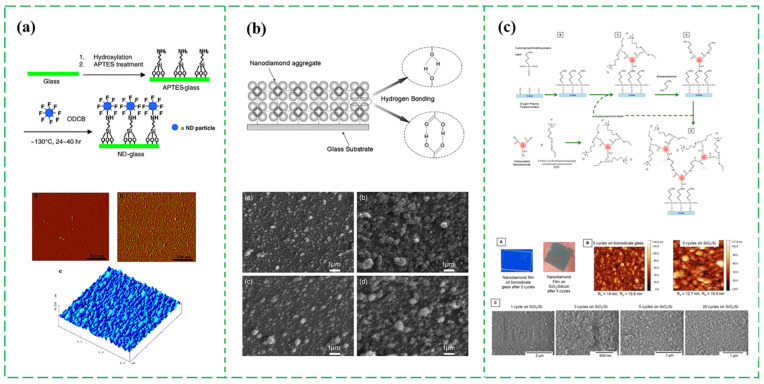
Typical production process and morphology images of ND films by assembling ND particles into continuous film [76,78,79]. (**a**) Reaction steps for coating glass with fluoro-ND and the AFM images of glass surface taken before and after coating [76]. (**b**) The formation process of ND films by SbS assembly and the SEM images of SbS films with 3 and 15 steps using ND dispersions with two different pHs [78]. (**c**) Reaction schematic process of directed covalent assembly of NDs, and the morphology and composition characterization of ND-COOH films [79].

Assembling ND particles into continuous film is an economically alternative solution to synthesize ND films, which can be well controlled the quality and surface roughness of ND film. In 2005, Liu Y. et al. first reported a mild wet chemistry coating process at low temperature for growing the fluorinated ND films [76], which was illustrated in the reaction steps of Figure 5a. ND clusters are linked with the glass surface through a robust covalent bonding. However, only one layer of ND could be coated. Soon after, Huang H. et al. found a facile process of synthesizing the ND thin films through drying ND dispersion aqueous at a relatively low temperature (<70 °C) and/or a sufficiently low pH (<4). The growth mechanism was ascribed that the hydrogen bonding interaction between the ND particles and the substrate under the directional convection induced by the water-evaporation flow [77]. Inspired by the above method and mechanism, Wang H.D. et al. used a step-by-step (SBS) assembling technique to synthesize a thicker ND film on glass side in 2012 [78]. Figure 5b shows the corresponding formation process and the obtained surface morphologies of ND films from 3 and 15 steps using ND dispersions with three pHs, where the hydrogen bonding is mainly accounted for the SbS assembly process. In addition, it can be seen that the surface morphology can be easily controlled and changed through adjusting the deposition steps. Compared with SbS films from 15 steps, the films from 3 steps had a denser structure with relatively smaller size ND particles. Moreover, the above reported thicker ND film are assembled relying on the weak van der Waal attractions with the poor mechanical properties and chemical stability. In 2020, Patoary N.H. et al. proposed a covalent assembly process of ND film on an amine-functionalized substrate through the cyclic attachment of the carboxylated ND and diamine linker [79], as shown in Figure 5c. The amide bonds formation and the diamine incorporation were contributed to the synthesis of ND films with similar ND grain size. Additionally, the assembled ND films exhibited a good mechanical integrity, a low inherent residual stress and a comparable thermal conductivity made by CVD. One years later (2021), the research group explored the effect of the pH variation using a low-ionic strength MES / KCl buffer and the thermal annealing process on the microstructure and thermal conductivity of the direct covalently assembled continuous ND films [80]. These results suggested that the buffer pH can be changed to adjust the surface morphology, film thickness, film apparent porosity, pore size distribution, and the thermal conductivity. Additionally, the thermal annealing temperature led to the aggregation of nanodiamond to segregated islands and increase the porosity of ND film. Therefore, more efforts should be conducted and explored for this method to reduce the ND aggregation and improve the smoothness of ND film.

### 2.3. Laser Irradiation

**Figure 6:**
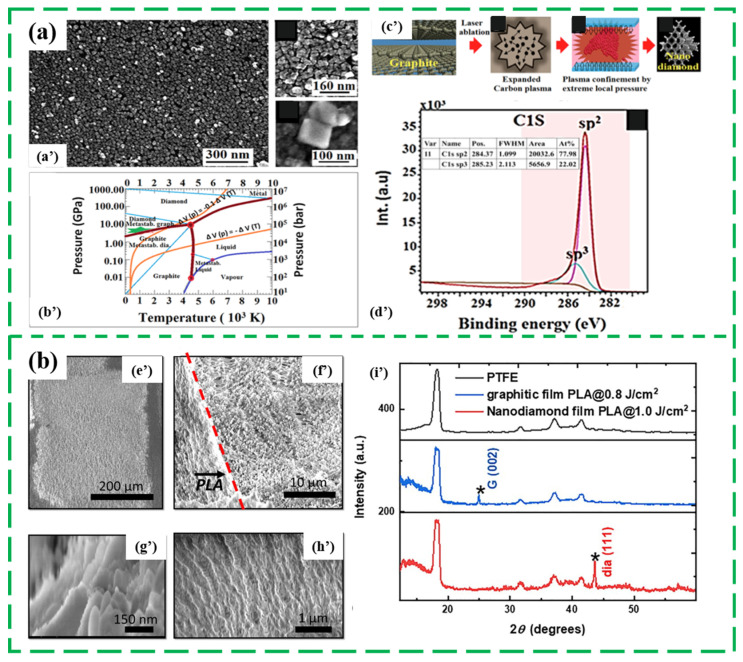
(**a**) The SEM images (**a’**), phase diagram (**b’**), schematic synthesis diagram (**c’**) and the deconvoluted C1s spectra (**d’**) of the ND films synthesized from graphite carbon [27], (**b**) The SEM images of laser patterned PTFE film (**e’**), irradiated nanodiamond region and the PTFE boundary (**f’**), liquid phase regrown nanodiamonds (**g’**), and, as acquired, PTFE (**h’**), and the XRD spectra of PTFE, PLA regrown diamond and graphitic films (**i’**) [81].

Laser irradiation is a novel method for synthesizing ND film with non-high temperature and non-high pressure. To date, many materials including graphite carbon, polytetrafluoroethylene (PTFE) was conducted to synthesize the ND films through the excimer pulsed laser annealing technology [27,28,81,82]. In 2014, Nian Q. et al. explored a confined pulsed laser deposition (CPLD) technique with the order of nanosecond to produce the patterned ND films from a layer of graphite topped with a glass cover sheet at room temperature and normal pressure [27]. As shown in Figure 6a, a dense ND film was converted from graphite in a matter of a few tens of nanoseconds with an laser intensity of about 5.8 GW/cm^2^, and 22% sp^3^-phase carbon elements were existed in the synthesized ND film. Additionally, the corresponding physical process can be concluded in three stages: (1) the graphite particles vapored into a dense plasma plume, (2) continuously heating and compressing of the plasma into the single carbon atoms and ions, (3) laser-induced high-temperature high-pressure plasma promotes the synthesis of ND films. 

Despite of the graphite carbon, PTFE, commercially known as the Teflon, are used as the carbon source in the formation of ND films [28,81]. Gupta S. et al. reported a direct argon fluoride excimer pulsed laser annealing (PLA) writing method for ND film with shorter duration (~100 ns) via melting PTFE in ambient condition in 2020, where the laser energy density is maintained at 1 J/cm^2^. Specially, the amorphous PTFE was firstly formed into undercooled molten carbon, then the molten carbon converted into <110> oriented diamond and ND film in the fast-quenching duration (~100 ns) in Figure 6b [81]. Subsequently, this group used the converted ND film resulted from the conversion of PTFE as the seeding layer to synthesize dense microdiamond coating in 2021 [82]. However, the smoothness of the synthesized ND films by laser irradiation method should be further studied and promoted.

## 3. Field Emission Properties of ND Film

Miniaturized electron cold-cathodes using nanomaterials nowadays attracted many attentions in the field emission devices, owing to the greatly improved field emission properties. Owing to low or negative electron affinity, stable chemistry, and high concentration of sp^2^ phase carbon, ND film is regarded as one of the most suitable field emission cold-cathode emitter nanomaterials to obtain low turn-on field (commonly at an electron emission density of 10 μA/cm^2^), high emitted current density at relatively lower applied field and long-term stability. Since Zhou D. et al. prepared a ND film by MWPECVD using CH_4_/N_2_ mixture gas, exhibiting an excellent performance with an onset field of 3.2 V/μm at 4 μA/cm^2^. It was found that the electronic gap state provided by the added N is beneficial to enhance the electron field emission at the surface of ND films [83]. Many attempts/changes were conducted for improving the field emission properties, such as adjusting the grain size and boundary, the concentration of sp^2^ phase, and doping type/level [84,85,86,87,88,89,90,91,92,93]. 

### 3.1. Adjusting Grain Size/Boundary and Internal Carbon Phase

**Figure 7:**
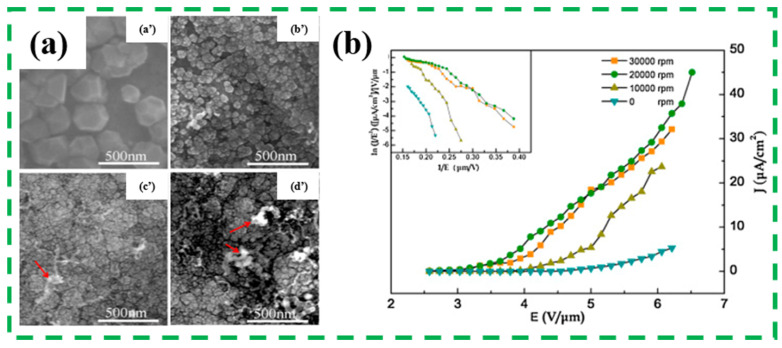
(**a**) The SEM images of the deposited ND film with different angular frequency ω of 0 rpm (**a’**), 10,000 rpm (**b’**), 20,000 rpm (**c’**), 30,000 rpm (**d’**). Additionally, (**b**) the corresponding *J*-*E* plots [88].

Reducing ND grain size and increasing grain boundaries and sp^2^ phase concentration are considered as the common approach of enhancing the field emission properties. For example, Wu K. et al. systematically investigated the effect of grain size on the field emission characteristics of ND thin films, and explained the reason of the enhanced field emission property based on the carbon structure of sp^2^ graphite/sp^3^ nanodiamond mixed phase [85]. It was proposed that sp^2^ graphite phase carbon conducted the channel between the substrate and film surface. Compared with the larger grain size of ND film, the smaller grain size of ND film was beneficial to emit electron from the whole surface and increase the emission site density. Wang S.G. et al. prepared an intrinsic ND film and a conventional CVD diamond film by MWPECVD method using CH_4_/H_2_ mixture [86]. The threshold electron field strength (*E*) of the as-synthesized intrinsic ND film emission was 4.0 V/μm at electron emission current density (*J*) of 1μA/cm^2^ and the maximum *J* ranged up to 560 μA/cm^2^ at *E* of 7.2 V/μm, which was much higher than the corresponding field emission properties of the conventional CVD diamond film It can be ascribed to the smaller size grains of ND film (15–20 nm), larger grain boundary, more sp^2^ phase non-diamond components, and defects incorporated into the films. Lee Y.C. et al. also found that the ND grain size and boundary can be adjusted by the growth conditions including the CH_4_/H_2_ ratio and the bias voltage of the MWPECVD system, which was deeply influenced the field emission characteristics [87]. Specially, A high emission *J* of 500 μA/cm^2^ and a low turn-on *E* of 8.5 V/μm was obtained from the prepared ND films deposited at a high bias voltage (–175 V) and a modest CH_4_/H_2_ ratio (5%:95%). Coincidentally, Long H. et al. studied the influence of periodic magnetic field (PMF) on the ND grain size/boundaries and internal content of sp^2^ phase as shown in Figure 7. It can be found from the SEM images that the nucleation density was enhanced and the crystal size was diminished by increasing the angular frequency ω of PMF. The fine grain size and continuously dense film can be easily obtained at a relatively high angular frequency, which was helpful to obtain an outstanding field emission performance with a turn-on field of 2.9 V/μm at 1μA/cm^2^, and a current emission density of 32.7 μA/cm^2^ at 6.5 V/μm [88].

### 3.2. Changing Doping Type/Level

Many researchers were attracted and joined into improving the field emission properties of ND films and further realizing the industrial field emission device application through the different doping type or level. Among them, N doping performs a significant role in enhancing the field emission performance due to the formation of the deep donor bandgap/defect states in the bandgap of ND film. For instance, the field emission properties of N-doped ND film prepared by Wang S.G. et al. exhibited a lower threshold *E* of 2.2 V/μm and larger maximum *J* of 720 μA/cm^2^ (at *E* = 6.4 V/μm), compared to the corresponding properties of intrinsic ND film. Additionally, it was attributed to the inducing the formation of the deep donation level and reduced work function by the N doping [86]. LeQuan X.C. et al. successfully incorporated N into ND film and reduced the turn-on *E* to 3.5 V/μm at 1 μA/cm^2^ [89]. The related results estimated that the nitrogen incorporation lowered the banding energy, increased the sp^2^ phase intensity and facilitated the electron escape. Recently, Guo X. et al. also proved the role of N doping for improving the properties. The turn-on *E* and the maximum *J* of the synthesized ND film with ~1.95 at.% N were, respectively, reduced to ~3.6 V/µm at 10 μA/cm^2^, and raise up to 1 mA/cm^2^ at 6 V/µm, and the excellent long-term emission stability can be achieved for the optimal N-doped ND film [15]. Therefore, it can be concluded that the introduction of N not just induces a n-type conductivity, but aggravates the formation of sp^2^ phase carbon, and thus enhances the field emission properties of ND film. 

**Figure 8:**
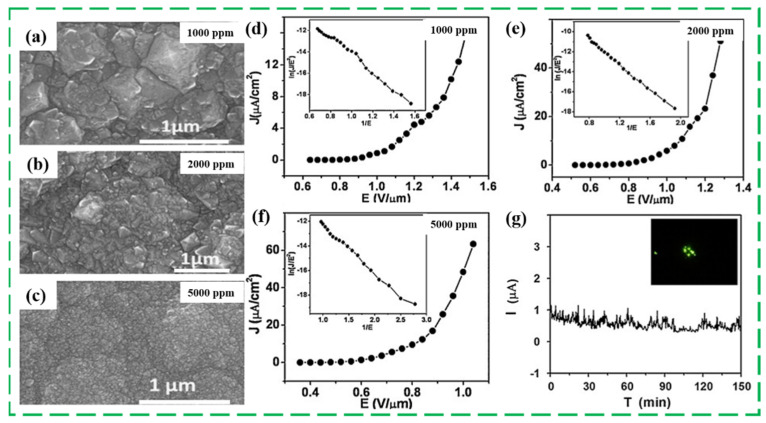
The SEM images (**a**–**c**), field emission curves (**d**–**f**) prepared with B concentrations of 1000 ppm, 2000 ppm, and 5000 ppm, and stability test (**g**) of B-doped ND films with B concentrations of 5000 ppm [90].

Apart from the N element, B is usually incorporated into the nanomaterial lattice to produce a p-type semiconductor. In 2005, Lee Y.C.’ group firstly conducted the production of B doping ND film and studied the effect of B doping amount on the field emission characteristics of ND films. The corresponding results showed that the field emission property was greatly improved with a proper concentration of B source, which was preliminarily attributed to the B induced aggregation of nanosized diamond and small diamond grain. However, it was suggested that the large amount of B species was presumably incorporated into the grain boundaries rather than into the lattice [17]. Koinkar P.M. et al. also developed the effect of the B_2_O_3_ concentration on the surface morphology and the field emission properties of NCD films [90]. The SEM images shown the influence of B_2_O_3_ concentration on the surface morphology. As the B_2_O_3_ concentration increased, the ‘crystal facets’ gradually disappeared, and the diameter size of ND particle reduced. In contrast to the field emission performance of Lee Y.C., the field emission properties of B-doped ND film monotonously enhanced with the increase in B concentration as shown in Figure 8. A low threshold field strength of 0.8 V/μm at ∼1 μA/cm^2^ for the samples prepared with 5000 ppm B_2_O_3_ concentration, and a stable long-term emission property at a preset value of ∼1 μA over a duration of 2 h were achieved, which was ascribed to the synergistic effect of smaller resistivity and nanodiamond size induced by the B addition. Additionally, the efficient electron emission sites is too few to be applied in the vacuum microelectronic devices and field emission devices. Moreover, the doped ND film is used to construct some promising field emission devices combining with the traditional field emission structure/materials (nanotips, nanotubes). The undoped and B-doped ND films coated 6H-SiC field emitter arrays (FEA) were synthesized by Ivanov O. A., and the B-doped ND film coated 6H-SiC FEA lower turn-on electric field, higher emission current, and long-term current stability than the undoped coated one and the original 6H-SiC FEA, because of the lower work function, higher conductivity and chemical inertness of B-doped ND films [91].

**Figure 9:**
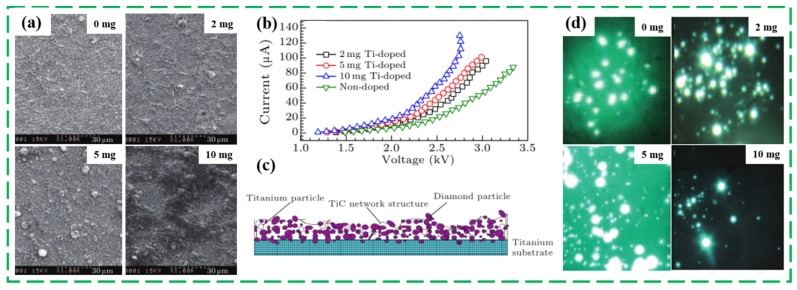
SEM images (**a**), field emission curves (**b**), microstructure model (**c**) and luminous photos (**d**) of the Ti-doped ND cathode film with different amounts of Ti powder, i.e., 0 mg, 2 mg, 5 mg, and 10 mg [92].

Additionally, metal is another comment doping material to improve the field emission properties of ND film, which can not only effectively lower the residual stress and modify the carbon films’ mechanical property, but also simultaneously improve the capability of emitting electrons. Yang Y. et al. proposed a titanium (Ti)-doped ND coating using electrophoresis and annealing (EPD @annealing) process, and studied the influence of Ti doping amount on the morphology, structure, and the field emission properties [92]. As exhibited in Figure 9a, as the amount of Ti powder increases, the irregular grain arrays are formed and increased. Additionally, the field emission measurement results in Figure 9b,d shown that the turn-on *E* decreased from 7.45 V/μm of intrinsic ND film to 5.95 V/μm of 10 mgTi-doped ND film, and the maximum *J* increased significantly from 35 μA/cm^2^ to 130 μA/cm^2^ at 13.8 V/μm, and the luminous point increased with a moderate increase in Ti amount powder, except for the 10 mg Ti-doped samples. Although the 10 mg Ti-doped ND film had the maximum emission current, a less luminous point and non-uniform brightness were achieved, implying that the comparatively large emission current is not necessary to obtain a good luminous effect. Additionally, the physical field emission enhancement mechanism of Ti-doped ND film was ascribed to the formed complicated microstructure (TiC buffer layer) greatly improved the electron transport capacity. Compared with the traditional emission materials, such as carbon nanotubes or carbon nanowalls, the number and uniformity of the luminous points are not satisfied and need to be further improved. Yang Y.N. group also conducted a comparative study about the effect of the Hf-doped and Ti-doped on the field emission properties of ND films, and found that the field emission characteristics of metal doped ND on Ti substrate were greatly influenced by the nature of metal Ti and Hf and the bonding reaction between nano-diamond and substrate Ti [93]. Recently, a moderate Ni-doped ND film was produced by Wang Y. et al. using EPD annealing process, in which the electron-rich Ni nano-powder improved the conductivity of ND coating and effectively promotes the conversion of diamond phase into high conductive graphite phase and thus resulting in an excellent EFE properties with turn on *E* of 1.38 V/μm and larger maximum *J* of 1323 μA/cm^2^ (at *E* = 2.94 V/μm) [30]. Importantly, the above studies suggest that the negative electron affinity might not be a prerequisite for emitting the electrons from the ND film surface. As discussed by Wu K. et al., the introduced graphitic channels provided a crucial pathways for enhancing the electrons emission [85]. Similarly, the density of grain boundaries, internal carbon phase, and the doping element in the ND films are considered to be the main reason for improving the field emission properties. The specific field emission mechanisms and the role of NEA at ND film surface should be further investigated and analyzed by more different advanced equipments (Table 2).

## 4. Conclusions and Prospect

The presented review is the overview of synthesis and the field emission properties of ND films. Fabricated methods are explained briefly, such as CVD technology, diamond seeding process, assembling NDs into continuous films, and laser irradiation technology. Additionally, the typical field emission properties of ND films are also reviewed, and the property improvement method are introduced and analyzed. It is clearly that the field emission properties are strongly promoted by adjusting the ND grain size/boundary, sp^2^ phase content and the doped type/level. 

In greater potentials, there will be more novel technologies in the future to improve the production and field emission performance of ND films. The problem that arises in the fabrication technology is the film quality and the production cost of intrinsic and doped ND films. On the one hand, although considerable researches on the synthesis of ND films have been conducted, the deposition rate, crystallinity, and the grain size uniformity are still required to seek more controllable and cost-effective ways. On the other hand, there is a large gap between the field emission properties of ND films and those of cone-shaped, fibrous or tubular nanomaterials, and the improvement methods of the number of thin film emission points and emission uniformity are still lack. In the future, the adjustment and optimization strategies of the morphology, work function, and conductivity of the ND film need to be further understood and controlled in the theoretical growth/emission mechanism research and experimental long-term emission test, so as to better guide the experimental realization of high-performance field emission devices.

## Figures and Tables

**Table 1:** Summary of the key growth parameters for the ND films by CVD with different energy source.

Gas Mixture	CVD	Growth Temperature (°C)	Growth Duration	Substrate Pretreatment	References
91%H_2_–9%CH_4_	DCCVD	800–950	30 min	Bias-enhanced nucleation (BEN)	[59]
90~98%H_2_^–^2~10%CH_4_	RFCVD	726.85–826.85	6–17 h	Scratched and seeding	[62]
98%~97%H_2_–2%~3% C_3_H_6_O	HFCVD	Filament: 2100; Substrate: 600–700	-	BEN	[64]
69%Ar–30%N_2_–1%CH_4_	MWCVD	400–1200	120 min	Scratched and seeding	[69]
7.5sccmH_2_–0~8sccmCO_2_–7.5sccmCH_4_	MWCVD	300	-	none	[70]
98.5%H_2_–0.5%O_2_–1%CH_4_	MWCVD	769–884	0–30 h	seeding	[72]
98.5%H_2_–1.5%CH_4_	HFCVD	Filament: 2100; Substrate: 800.	7 h	Bias-enhanced nucleation (BEN)	[73]
99.4%H_2_–0.6%CH_4_ (B_2_H_6_/CH_4_ ratio of 5%)	HFCVD	Filament: 2200; Substrate: 800.	40 min	seeding	[74]
93%,97%H_2_–7%,3%CH_4_	HFCVD	-	-	Electrochemically treated and seeding	[75]

**Table 2:** The field emission properties of intrinsic/doping ND films in recent reports.

Gas Mixture (Method)	Sample	Grain Size	Turn-On /Threshold Electron Field		Maximum Current Density		References
			E	J	J_max_	E	
4%CH_4_/ 96%N_2_ (CVD)	N-doped ND film	10–30 nm	3.2 V/μm	4 μA/cm^2^	400 μA/cm^2^	6 V/μm	[83]
20%CH_4_/ 80%H_2_ (CVD)	ND film	10 nm	2.5 V/μm	10 μA/cm^2^	150 μA/cm^2^	~3.75 V/μm	[84]
1%CH_4_/ 99%H_2_ (CVD)	ND film	15–20 nm	4.0 V/μm	1 μA/cm^2^	560 μA/cm^2^	7.2 V/μm	[86]
1%CH_4_/ 4%H_2_ /95%N_2_ (CVD)	N-doped ND film	15–20 nm	2.2 V/μm	1 μA/cm^2^	720 μA/cm^2^	6.4 V/μm	[86]
5%CH_4_/ 95%H_2_ (CVD)	ND film	20 nm	8.5 V/μm	10 μA/cm^2^	500 μA/cm^2^	20 V/μm	[87]
1%CH_4_/ 99%H_2_ (CVD)	ND film	~	2.9 V/μm	1 μA/cm^2^	32.7 μA/cm^2^	6.5 V/μm	[88]
9.1%CH_4_/ 81.8% H_2_/9.1%N_2_ (CVD)	N-doped ND film	10–20 nm	3.5 V/μm	1 μA/cm^2^	-	-	[89]
5%CH_4_/ 94.5%H_2_/0.5%B(OCH_3_)_3_ (CVD)	B-doped ND film	20 nm	18 V/μm	10 μA/cm^2^	700 μA/cm^2^	30 V/μm	[17]
19.9%CH_4_/ 79.6%H_2_/0.5%B_2_O_3_ (CVD)	B-doped ND film	<30 nm	0.8 V/μm	1 μA/cm^2^	~60 μA/cm^2^	~1 V/μm	[91]
1.92%CH_4_/ 98%H_2_/0.08%B(OCH_3_)_3_ (CVD)	B-doped ND film coated 6H-SiC FEA	-	9 V/μm	1 μA/cm^2^	~50 μA/cm^2^	~16.2 V/μm	[92]
Glucose@urea solid layer (heating precursor)	N-doped ND film	20~100 nm	3.6 V/μm	10 μA/cm^2^	1000 μA/cm^2^	6.0 V/μm	[15]
ND powder/Ti powder (EPD @annealing)	Ti-doped ND coating	-	5.95 V/μm	1 μA/cm^2^	130 μA/cm^2^	13.8 V/μm	[93]
ND powder/Ni nano powder (EPD @annealing)	Ni-doped ND film	-	1.38 V/μm	1 μA/cm^2^	1323 μA/cm^2^	2.94 V/μm	[30]
